# Breast Cancer in Chinese Females Aged 25 Years and Younger

**DOI:** 10.1155/2021/4891936

**Published:** 2021-11-30

**Authors:** Lixi Li, Dan Lv, Jingtong Zhai, Di Zhang, Xiuwen Guan, Fei Ma

**Affiliations:** Department of Medical Oncology, National Cancer Center/National Clinical Research Center for Cancer/Cancer Hospital, Chinese Academy of Medical Sciences, Peking Union Medical College, Beijing 100021, China

## Abstract

**Background:**

Breast cancer has both aggressive clinicopathological characteristics and a poor prognosis in young females. However, limited information is available for breast cancer in Chinese females aged ≤25 years. Therefore, we aimed to explore prognostic factors for invasive disease-free (iDFS) and overall survival (OS) among breast cancer patients aged ≤25 years.

**Methods:**

We retrospectively analyzed data from 174 Chinese females aged ≤25 years with invasive breast cancer treated in the Cancer Hospital of the Chinese Academy of Medical Sciences from January 1, 1999, to December 31, 2018. Univariate and multivariate Cox regression analyses were performed to identify independent prognostic factors.

**Results:**

The median follow-up time was 75 months (ranging from 1 to 236 months). Breast cancer patients aged ≤25 years exhibited aggressive clinicopathological characteristics, including advanced tumor stage (21.8%), lymph node metastasis (47.1%), lymphovascular invasion (24.1%), estrogen receptor negativity (44.3%), progesterone receptor (PR) negativity (42.5%), and triple-negative breast cancer (25.3%). Among them, 50 cases had locoregional recurrence and metastasis, 20 had bilateral invasiveness, and 33 had breast cancer-specific deaths. Cox multivariate analysis identified that diagnosis delay, PR status, and radiotherapy were significant prognostic factors for both iDFS and OS (*P* < 0.05). The risk of recurrence and metastasis was five times higher in N3 than in N0 (HR: 6.778, 95% CI: 2.268–17.141, *P* < 0.001). Patients with lymphovascular invasion had a threefold increase in the risk of breast cancer-specific death (HR: 4.217, 95% CI: 1.956–9.090, *P* < 0.001). No differences were observed between mastectomy and breast-conserving surgery (BCS) plus radiotherapy for iDFS or OS (iDFS: *χ*^2^ = 0.678, *P*=0.410; OS: *χ*^2^ = 0.165, *P*=0.685).

**Conclusions:**

Breast cancer in females ≤25 years old was associated with aggressive clinical features and a worse prognosis. Young females with breast lumps should receive timely diagnosis and treatment. Young breast cancer patients with lymphovascular invasion, PR-negative status, and lymph node metastasis have an increased risk of experiencing recurrence and metastasis and should hence be closely monitored. Age at diagnosis should not be the sole deciding factor for surgical treatment methods.

## 1. Background

Breast cancer is the most common cancer in females worldwide, with the incidence rate among females aged 20–29 years increasing by nearly 2% annually [1]. In China, the median age of breast cancer at diagnosis is around 50 years, which is 10 years older than the average in the European Union and the United States [2]. Nevertheless, there are breast cancer patients aged ≤25 years known as very young breast cancer (VYBC) patients. The incidence of VYBC ranges from 0.4% to 1.2% [3–6]. Furthermore, the threshold for age at diagnosis for young breast cancer has remained controversial. Most studies define breast cancer in females younger than 35 or 40 years old as young breast cancer [7–12]. However, information about breast cancer in Chinese females aged ≤25 years is limited. VYBC has aggressive clinical and pathological features and is more likely to develop tumors of a larger size, have higher lymph node positivity rates, present with more advanced stages, have increased lymphovascular invasion, have higher histological grades, have lower hormone receptor positivity rates, have overexpression of the human epidermal growth factor receptor 2 (HER2), and have a higher proportion of triple-negative breast cancer (TNBC) [5, 6, 13]. Furthermore, breast cancer in young females, particularly those aged ≤25 years, has been associated with a worse prognosis. Some studies suggest that aggressive clinicopathological features are significant prognostic factors for VYBC [3, 5, 13]. Furthermore, a small sample retrospective analysis identified that diagnostic delay of >3 months is a prognostic factor for overall survival (OS) [13].

This report is a retrospective study that aimed to explore the prognostic factors for invasive disease-free survival (iDFS) and OS in breast cancer patients aged ≤25 years.

## 2. Methods

### 2.1. Study Populations

Females aged ≤25 years diagnosed with invasive breast cancer who visited National Cancer Center from January 1, 1999, to December 31, 2018, were recruited in this study. The tumor node metastasis (TNM) staging system was classified according to the American Joint Committee on Cancer (8th edition). A total of 174 patients were included. The study was approved by the ethics committee of our institution, and written informed consent was signed by all of the patients.

### 2.2. Definition of Endpoint Events

We defined iDFS as the period of time from surgery to locoregional recurrence, invasive ipsilateral breast cancer, invasive contralateral breast cancer, or distant metastasis. We defined OS as the period of time from surgery to death from any cause or the last follow-up visit. To analyze the prognostic factors for iDFS and OS, stage IV breast cancer was not included in either analysis.

### 2.3. Statistical Methods

Descriptive statistics were obtained, and Pearson's *χ*^2^ test was used to estimate *P* values. The iDFS and OS were assessed using Kaplan–Meier estimates. The Breslow test was used to assess the equality of the survivor function across groups. The Cox proportional hazards model was constructed to adjust for possible confounding factors and used in an analysis that included all factors with values of *P* < 0.2 in univariate analysis. However, the TNM stage and molecular subtypes have a collinear relationship with the other variables and were hence excluded from the multivariate analysis. All statistical tests were two-sided, and *P* values < 0.05 were considered significant. All statistical analyses were performed using SPSS version 24.0 (SPSS Inc., Chicago, IL, USA).

## 3. Results

### 3.1. Clinical Characteristics

A total of 174 breast cancer patients aged ≤25 years diagnosed at our institution were recruited, corresponding to 1.11% of all breast cancer cases in the same period. The age at diagnosis ranged from 18 to 25 years. Patients' clinicopathological characteristics are shown in Table 1. According to our findings, 50 (28.7%) patients were diagnosed with a delay of ≥6 months. There were 25 cases of pregnancy-associated breast cancer (PABC), 7 (28.0%) during pregnancy and 18 (72.0%) within one year after delivery. Nearly half (49.4%) of the patients had an age at menarche ≥14 years. Moreover, 73% of patients had not been pregnant before the diagnosis, and 78.7% (37/47) of pregnant patients had experienced at least one miscarriage. Of the 174 participants (family history available), 17 (9.8%) had at least one first- or second-degree relative diagnosed with breast or ovarian cancer. Furthermore, 11 (6.3%) patients had bilateral breast cancer, of whom 10 were diagnosed with metachronous bilateral breast cancer ≥6 months after the diagnosis of the first primary breast cancer and 1 had simultaneous bilateral breast cancer diagnosed <6 months after the diagnosis of the first primary breast cancer. A higher proportion of young breast cancer patients underwent breast-conserving surgery (BCS) (97/174, 55.7%), of whom 87 (89.7%) received radiotherapy than mastectomy (72/174, 41.4%). The main histological subtypes were invasive ductal carcinoma (153/174, 87.9%), medullary carcinoma (8/174, 5.5%), invasive lobular carcinoma (4/174, 2.3%), mucinous carcinoma (4/174, 2.3%), cribriform carcinoma (2/174, 1.1%), papillary carcinoma (2/174, 1.1%), and secretory carcinoma (1/174, 0.6%). A total of 38 (21.8%) patients were diagnosed in the advanced stages (stages III and IV), and 5 were diagnosed at stage IV. The proportion of lymphovascular invasion was 24%. Estrogen receptor (ER) was positive in 55.7% (97/174) of patients, progesterone receptor (PR) was positive in 57.5% (100/174) of patients, and HER2 was overexpressed in 24.1% (42/174) of patients. Luminal B was the most common molecular subtype, whereas the TNBC and HER2 subtypes accounted for 25.3% and 13.2% of the total number of cases, respectively. From the start of follow-up to the cut-off date, 50 patients had recurrence and metastasis and 33 patients died of breast cancer.

### 3.2. Univariable and Multivariable Analyses of iDFS

**Figure 1:**
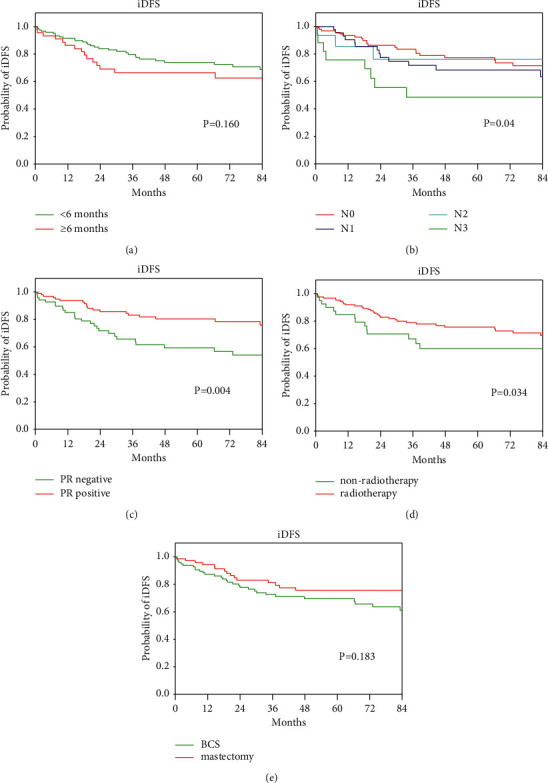
Invasive disease-free survival analysis of very young breast cancer patients. (a) Diagnosis delay ≥6 months versus diagnosis delay <6 months; (b) lymph node: N0 versus N1 versus N2 versus N3; (d) received radiotherapy versus not received radiotherapy; (e) mastectomy versus breast-conserving surgery.

The 1-, 3-, and 5-year iDFS rates for VYBC were 90.2%, 76.1%, and 71.9%, respectively. According to the univariate analysis, tumor size, lymph node metastasis, lymphovascular invasion, ER status, PR status, and radiotherapy were significant factors for iDFS (*P* < 0.05) (Table 1). Patients with large tumors, high lymph node metastasis stages, lymphovascular invasion, ER-negative status, PR-negative status, and not receiving radiotherapy were more likely to experience relapse and metastasis. Moreover, diagnosis delay, PABC, age at menarche, family history of breast or ovarian cancer, histological type, surgical methods, and lymph node dissection methods were not associated with iDFS (*P* > 0.05) (Table 1). The results of Cox multivariate analysis showed that diagnosis delay, lymph node metastasis, PR status, radiotherapy, and surgical methods were the most significant prognostic factors for iDFS (Table 2). Diagnosis delay ≥6 months, ER-negative status, and lymph node metastasis were identified as risk factors for recurrence and metastasis in VYBC. The risk of recurrence and metastasis for patients with lymph node metastasis N3 stage was over five times higher than that for patients with N0 stage (no lymph node metastasis) (HR: 6.778, 95% CI: 2.680–17.141, *P* < 0.001) (Table 3). Mastectomy significantly improved iDFS compared with BCS (HR: 0.190, 95% CI: 0.090–0.402, *P* < 0.001) (Figure 1) as did receiving radiotherapy (HR: 0.181, 95% CI: 0.085–0.383, *P* < 0.001) (Figure 1).

### 3.3. Univariable and Multivariable Analyses of OS

**Figure 2:**
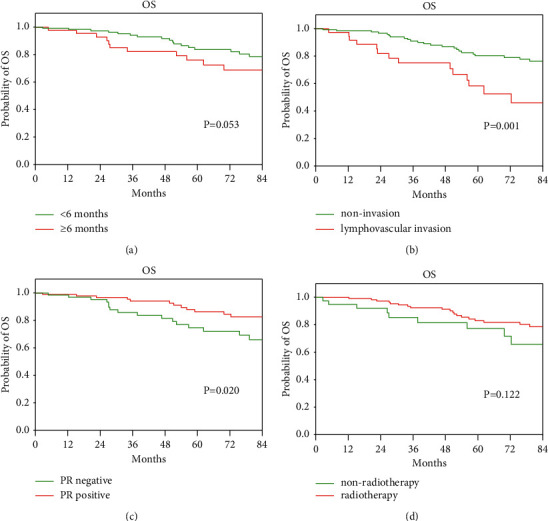
Overall survival analysis in very young breast cancer patients. (a) Diagnosis delay ≥6 months versus diagnosis delay <6 months; (b) lymphovascular invasion versus noninvasion; (c) PR-positive status versus PR-negative status; (d) radiotherapy versus nonradiotherapy.

The 1-, 3-, and 5-year OS rates for VYBC were 98.8%, 90.7%, and 81.6%, respectively. The median OS was not reached. According to the univariate analysis, tumor size, lymph node metastasis, vascular tumor thrombus, and PR status were significant factors of OS (*P* < 0.05) (Table 2). Patients with large diameter tumors, higher stage of lymph node metastasis, lymphovascular invasion, and PR-negative status had a higher risk of breast cancer-specific mortality (*P* < 0.05). The results of the Cox multivariate analysis showed that diagnosis delay, lymphovascular invasion, PR status, and radiotherapy were significant prognostic factors for OS (*P* < 0.05) (Table 4; Figure 2). Age at menarche, pregnancy, abortion, PABC, breast or ovarian cancer family history, tumor size, lymph node stage, ER and HER2 status, surgical methods, lymph node surgical methods, and histological subtypes were not associated with OS in VYBC (*P* > 0.05).

### 3.4. Mastectomy versus BCS Combined with Radiotherapy

**Figure 3:**
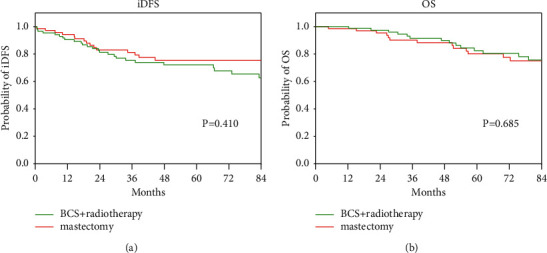
Kaplan–Meier curves of breast-conserving surgery (BCS) plus radiotherapy versus mastectomy. (a) Invasive disease-free survival (iDFS); (b) overall survival (OS); no difference in iDFS and OS was observed between BCS plus radiotherapy and mastectomy.

According to the univariate analysis for iDFS, patients who underwent BCS had worse survival than those who underwent mastectomy. However, most patients who underwent BCS (87/97, 89.7%) received radiotherapy. In Cox multivariate analysis for iDFS and OS, the radiotherapy group had a better prognosis than the nonradiotherapy group. We further analyzed the effects of mastectomy and BCS combined with radiotherapy on the prognosis of young breast cancer patients and found no differences in iDFS and OS between mastectomy and BCS combined with radiotherapy (iDFS: *χ*^2^ = 0.678, *P*=0.410; OS: *χ*^2^ = 0.165, *P*=0.685) (Figure 3).

## 4. Discussion

The incidence of VYBC is low, and only few studies have explored the prognostic factors for VYBC. Young breast cancer has aggressive clinicopathological characteristics and a worse prognosis [14]. The results of this study showed that a greater proportion of young breast cancer patients had ER- and PR-negative status, TNBC status, lymphovascular invasion, lymph node positive status, and more advanced stages. Studies have confirmed that lymph node metastasis and diagnostic delay of >3 months are prognostic factors for breast cancer among females ≤25 years old [5, 13]. By adjusting for confounding factors, our study identified that VYBC patients with lymph node metastasis and diagnostic delay of ≥6 months had a worse prognosis, which is consistent with the results of previous studies. Furthermore, lymphovascular invasion, PR status, radiotherapy, and surgical methods were associated with VYBC prognosis. These results have not yet been reported in previous studies because of the unavailability of a large number of patients. Therefore, adjuvant therapy for VYBC patients with one or more risk factors should be strengthened.

Asian females have a higher proportion of dense breast tissue than Western females, and young Chinese females usually have denser and smaller-sized breasts [15]. Dense breast tissue reduces the sensitivity of mammography and ultrasound to the mass; hence, small breast lumps are easily overlooked, and the time to diagnosis and treatment often become delayed in young breast cancer. In our study, nearly half of all patients experienced a diagnosis delay of ≥6 months, which was associated with worse iDFS and OS. PABC patients often had a significantly higher risk of death than non-PABC patients [16]. In our study, 25 of 174 young breast cancer patients were diagnosed with PABC, but there were no differences between PABC and non-PABC patients concerning iDFS and OS. Furthermore, pregnancy and abortion were not associated with the prognosis of young breast cancer patients. It is noteworthy that most young breast cancer patients were unmarried and childless. A meta-analysis revealed that females who received systemic therapy after surgery had a 14% chance of falling pregnant (overall pooled estimate), but the pregnancy rate was 3% [17]. According to the ESO-ESMO 4th International Consensus Guidelines for Breast Cancer in Young Women, young breast cancer patients requiring fertility interventions should receive fertility preservation strategies as soon as possible [9]. A large number of retrospective studies have confirmed that pregnancy has no adverse effect on the prognosis of breast cancer [18].

The proportion of young Chinese females undergoing BCS is higher than that of elderly females [12]. Moreover, the proportion of VYBC patients who received BCS in this study is higher than those who received mastectomy. In contrast, total mastectomy was the most common initial surgical procedure in females ≤25 years old in Western countries [5]. This may be because both guideline recommendations and female perceptions regarding the appearance of breasts differ across countries. Comparative randomized trials have shown equivalent survival for mastectomy and BCS plus radiation [19]. However, more studies have reported that BCS for young breast cancer is associated with a higher risk of local recurrence [20–22], which is consistent with our results. The NCCN guidelines recommend that young breast cancer patients should be cautious about BCS. To further explore the impact of surgical methods on the prognosis of breast cancer among females ≤25 years old, we analyzed the iDFS and OS in both mastectomy and BCS plus radiotherapy. Interestingly, no significant difference was observed. A systematic meta-analysis confirmed that BCS plus radiation and mastectomy led to comparable survival in females <40 years old with early-stage breast cancer [23]. Nevertheless, young breast cancer has more aggressive clinical features than older breast cancer; therefore, it is inappropriate to directly compare the effects of surgical methods on prognosis.

Similarly, axillary lymph node dissection is not significantly associated with the prognosis of young breast cancer patients. Therefore, it is recommended that BCS and sentinel lymph node biopsy be the first choice for young patients who meet the indications. Furthermore, radiotherapy can significantly improve iDFS and OS in young breast cancer patients. Age at diagnosis alone is not a suitable reason to choose mastectomy and axillary lymph node dissection, but the indications for radiotherapy can be appropriately relaxed in young breast cancer patients.

This retrospective study analyzed the prognostic factors for iDFS and OS in VYBC, with the advantage of a relatively large sample of females ≤25 years old and long-term follow-up data. This study confirmed that young breast cancer has unique clinicopathological characteristics and clarified the prognostic factors for VYBC, thereby providing an evidence-based medical basis for guiding diagnosis and treatment. However, to further study the unique biological behavior of young breast cancer, research studies concerning mechanisms are indispensable.

## 5. Conclusion

Breast cancer in females ≤25 years old was associated with aggressive clinical features and a worse prognosis. Therefore, young females with breast lumps should receive timely diagnosis and treatment. Young breast cancer patients with lymphovascular invasion, PR-negative status, and lymph node metastasis have an increased risk of experiencing recurrence and metastasis and should be closely monitored. Furthermore, the age at diagnosis should not be the sole reason for decisions made regarding the surgical method for treatment.

## Figures and Tables

**Table 1:** Clinicopathological characteristics of young breast cancer patients at initial diagnosis.

Characteristics	Total (*n* = 174)	%
Age (average ± SD)	23.62 ± 1.73	—
*Diagnosis delay*		
＜6 months	124	71.3
≥6 months	50	28.7

*Menarche*		
<14 years	88	50.6
≥14 years	86	49.4

*Pregnancy*		
Yes	47	27.0
No	127	73.0

*Abortion*		
Yes	37	21.3
No	137	78.7

*Family history*		
Yes	17	9.8
No	157	90.2

*PABC*		
Yes	25	14.4
No	157	85.6

*Histological subtypes*		
IDC	153	87.9
Medullary	8	4.6
ILC	4	2.3
Papillary	4	2.3
Others	5	2.9

*Tumor size*		
T1	93	53.4
T2	71	40.8
T3	6	3.4
T4	4	2.3

*Lymph node*		
N0	92	52.9
N1	45	25.9
N2	15	8.6
N3	22	12.6

*Metastasis*		
M0	169	97.1
M1	5	2.9

*TNM stage*		
I	56	32.1
II	80	46.0
III	33	19.0
IV	5	2.9

*Lymphovascular invasion*		
Yes	42	24.1
No	132	75.9

*ER*		
Positive	97	55.7
Negative	77	44.3

*PR*		
Positive	100	57.5
Negative	74	42.5

*HER2*		
Positive	42	24.1
Negative	132	75.9

*Molecular subtype*		
Luminal A	19	10.9
Luminal B (HER2-)	69	39.7
Luminal B (HER2+)	19	10.9
HER2 positive	23	13.2
TNBC	44	25.3

*Surgery*		
Mastectomy	72	41.4
Conservation	97	55.7
Palliative	2	1.1
No	3	1.7

*SLN/ALND*		
SLN	49	28.2
ALND	120	71.8
Radiotherapy		
Yes	129	74.1
No	45	25.9

**Table 2:** Results of univariate analysis of risk factors for iDFS and OS in young breast cancer patients.

Variable	Total (*n* = 169)	Recurrence/metastasis		*χ*2	*P*	Death		*χ*2	*P*
		Yes (*n* = 50)	No (*n* = 119)			Yes (*n* = 33)	No (*n* = 136)		
*Diagnosis delay*									
<6 months	123	33(66.0%)	90(75.6%)	1.970	0.160	19(57.6%)	104(76.5%)	3.747	0.053
≥6 months	46	17(34.0%)	29(22.4%)			14(42.4%)	32(23.5%)		

*Menarche*									
<14 years	87	29(58.0%)	58(48.7%)	0.216	0.642	17(51.5%)	70(51.5%)	0.994	0.319
≥14 years	82	21(42.0%)	61(51.3%)			16(48.5%)	66(48.5%)		

*Pregnancy*									
Yes	43	13(26.0%)	30(25.2%)	0.265	0.607	8(24.2%)	35(25.7%)	0.170	0.680
No	126	37(74.0%)	89(74.8%)			25(75.8%)	101(74.3%)		

*Abortion*									
Yes	33	7(14.0%)	26(21.8%)	0.425	0.514	5(15.2%)	28(20.6%)	0.103	0.748
No	136	43(86.0%)	93(78.2%)			28(84.8%)	108(79.4%)		

*Family history*									
Yes	17	46(7.8%)	106(5.9%)	0.742	0.389	3(9.1%)	14(10.3%)	0.018	0.892
No	152	4(92.2%)	13(94.1%)			30(90.9%)	122(89.7%)		

*PABC*									
Yes	22	5(10%)	17(14.3%)	0.081	0.776	5(15.2%)	17(12.5%)	0.372	0.542
No	149	45(90%)	102(85.7%)			28(84.8%)	121(87.5%)		

*Histological subtypes*									
IDL	148	45(90%)	103(86.6%)	0.875	0.350	31(93.9%)	117(86.0%)	0.996	0.318
Others	21	5(10%)	16(13.4%)			2(6.1%)	19(14.0%)		

*Tumor size*									
T1	93	25(31.1%)	68(48.5%)	8.695	0.013	15(45.5%)	78(57.4%)	9.529	0.009
T2	69	22(54.4%)	47(46.6%)			15(45.5%)	54(39.7%)		
T3 and T4	7	3(8.7%)	4(4.4%)			3(9.1%)	4(2.9%)		

*Lymph node*									
N0	92	23(30.1%)	69(57.4%)	8.287	0.04	14(42.4%)	78(57.4%)	11.732	0.008
N1	45	14(31.1%)	31(27.9%)			10(30.3%)	35(25.7%)		
N2	15	4(16.5%)	11(10.3%)			2(6.1%)	13(9.6%)		
N3	17	9(22.3%)	8(4.4%)			7(21.2%)	10(7.4%)		

*Lymphovascular invasion*									
Yes	37	14(28.0%)	23(19.3%)	5.014	0.025	12(36.4%)	25(18.4%)	11.285	0.001
No	132	36(72.0%)	96(80.7%)			21(63.6%)	111(81.6%)		

*ER*									
Positive	96	22(42.0%)	74(62.2%)	5.106	0.024	14(42.2%)	82(60.3%)	3.292	0.070
Negative	73	28(56.0%)	45(37.8%)			19(57.6%)	54(39.7%)		

*PR*									
Positive	99	21(42.0%)	78(65.5%)	8.119	0.004	13(39.4%)	86(63.2%)	5.450	0.020
Negative	70	29(58.0%)	41(34.5%)			20(60.6%)	50(36.8%)		

*HER2*									
Positive	38	11(22.0%)	27(22.7%)	0.873	0.350	7(21.2%)	31(22.8%)	0.669	0.413
Negative	131	39(78.0%)	92(77.3%)			26(78.8%)	105(77.2%)		

*Surgery*									
Mastectomy	72	18(36.0%)	54(45.4%)	1.775	0.183	15(45.4%)	57(41.9%)	0.136	0.712
BCS	97	32(64.0%)	65(54.6%)			18(54.5%)	79(58.1%)		

*SLN/ALND*									
SLN	49	10(20%)	39(32.8%)	2.552	0.110	6(18.2%)	43(31.6%)	0.450	0.502
ALND	120	40(80%)	80(67.2%)			27(81.8%)	93(68.4%)		

*Radiotherapy*									
Yes	129	34(68.0%)	95(79.8%)	4.491	0.034	23(69.7%)	106(77.9%)	2.387	0.122
No	40	16(32.0%)	24(20.2%)			10(30.3%)	30(22.1%)		

**Table 3:** Results of the Cox multivariate analysis of risk factors for iDFS.

Variable	*β*	HR	95% CI	*P*
Diagnosis delay (<6 months ref)		1		
≥6 months	0.665	1.944	1.016∼3.722	0.045
Lymph node (N0 ref)		1		
N1	0.784	2.191	1.084∼4.428	0.029
N2	1.468	4.341	1.349∼13.968	0.014
N3	1.914	6.778	2.268∼17.141	<0.001
PR (PR negative ref)		1		
PR positive	−1.284	0.277	0.148∼0.518	<0.001
Radiotherapy (nonradiotherapy ref)		1		
Radiotherapy	−1.712	0.181	0.085∼0.383	<0.001
Surgery (conservation ref)		1		
Mastectomy	−1.662	0.190	0.090∼0.402	<0.001

**Table 4:** The results of the Cox multivariate analysis of risk factors for OS.

Variable	*β*	HR	95% CI	*P*
Diagnosis delay (<6 months ref)		1		
≥6 months	0.922	2.513	1.235∼5.116	0.011
Lymphovascular invasion (noninvasion ref)		1		
Invasion	1.439	4.217	1.956∼9.090	<0.001
PR (PR negative ref)		1		
PR positive	−1.198	0.302	0.146∼0.624	0.001
Radiotherapy (nonradiotherapy ref)		1		
Radiotherapy	−1.251	0.286	0.119∼0.689	0.005

## Data Availability

The data supporting the conclusions of this article are included within the article.

## Abbreviations

iDFS: Invasive disease-free survival
OS: Overall survival
ER: Estrogen receptor
PR: Progesterone receptor
HER2: Human epidermal growth factor receptor 2
BCS: Breast-conserving surgery
VYBS: Very young breast cancer
TNBC: Triple-negative breast cancer
TNM: Tumor node metastasis
PABC: Pregnancy-associated breast cancer.
